# Effect of growth hormone therapy on liver enzyme and other cardiometabolic risk factors in boys with obesity and nonalcoholic fatty liver disease

**DOI:** 10.1186/s12902-022-00967-y

**Published:** 2022-02-26

**Authors:** Jiang Xue, Shuang Liang, Jiahui Ma, Yanfeng Xiao

**Affiliations:** 1 Department of Pediatrics, The Second Hospital, Cheeloo College of Medicine, Shandong University, Jinan, China; 2 Department of Pediatrics, The Second Affiliated Hospital of Xi’an Jiaotong University, Xi’an, China; 3 Department of Cardiology, Yankuang New Journey General Hospital, Zoucheng, China

**Keywords:** Growth hormone, Therapy, Insulin like growth factor 1, Non-alcoholic fatty liver disease, Liver enzyme

## Abstract

**Background:**

Non-alcoholic fatty liver disease (NAFLD) has become the most common causes of liver disease in children and adolescents. Although several reports have confirmed the significant correlation between NAFLD and growth hormone (GH)-insulin-like growth factor 1(IGF-1) axis, no study further investigates whether or not recombinant human GH (rhGH) treatment can improve NAFLD in obese children.

**Methods:**

This study was a randomized, open-label study comprising 44 boys with obesity and NAFLD (11.76 ± 1.67 year) to evaluate the effects of 6 months of rhGH administration for boys with obesity and NAFLD. The subjects were randomized divided into treatment group (subjects with recombinant human GH (rhGH)) and control group for 6 months.

**Results:**

After 6 months, IGF-1 increased significantly during rhGH treatment, in comparison with the control group (582.45 ± 133.00 vs. 359.64 ± 129.00 ng/ml; *p* < 0.001). A significant reduction in serum alanine aminotransferase(ALT) (15.00 vs. 28.00 U/L; *p* = 0.001), aspartate aminotransferase(AST) (20.00 vs. 24.50U/L; *p* = 0.004), gamma glutamyl transferase(GGT) (14.50 vs. 28.50 U/L; *p* < 0.001) was observed in the GH-treated boys. In addition, the rhGH group showed a significant decrease in C reactive protein (CRP) (1.17 ± 0.76 vs. 2.26 ± 1.43 mg/L) and body mass index standard deviation scores (BMI SDS) (2.28 ± 0.80 vs. 2.71 ± 0.61) than the control group (*p* = 0.003, *p* = 0.049 respectively). GH treatment also reduced low density lipoprotein cholesterol (LDL-C) (2.19 ± 0.42 vs. 2.61 ± 0.66 mmol/L; *p* = 0.016) and increased high density lipoprotein cholesterol (HDL-C) (1.30 vs. 1.15 mmol/L; *p* = 0.005), and there were no changes in total cholesterol (TC), triglycerides (TG) and uric acid(UA) between the treatment group and the control group.

**Conclusion:**

Our findings suggest that 6 months treatment with rhGH may be beneficial for liver enzyme and can improve obesity-related other cardiovascular and metabolic complications in boys with obesity and NAFLD.

## Background

As one of the complications of obesity, non-alcoholic fatty liver disease (NAFLD) has become the one of the most common causes of liver disease in children and adolescents [1], which seriously threatens the human health. At present, weight management is the only established treatment for NAFLD in children and thus it is the primary treatment recommendation in guidelines from the North American Society for Pediatric Gastroenterology, Hepatology and Nutrition and the American Gastroenterological Association [2, 3]. But it is difficult to achieve that in children. Thus, it is important and necessary to better understand the pathophysiology of NAFLD and to find new targets for the treatment of NAFLD in obese children and adolescents.

In recent years, several reports have confirmed the significant correlation between NAFLD and growth hormone (GH)-Insulin-like growth factor 1(IGF-1) axis. On the one hand, prior studies have suggested that there was a potential interaction between GH deficiency and NAFLD in adolescents and adults [4–6], and replacement therapy with recombinant human GH (rhGH) has demonstrated favorable effects in GH deficiency patients due to the fact that it often significantly improves the hepatic steatosis and fibrosis [4–8]. On the other hand, in adults with normal pituitary function, researchers also found that NAFLD patients had significant reductions in GH and IGF-1 levels, and the deficiencies in GH and IGF-1 contributed to the occurrence and development of NAFLD [9, 10]. In addition, animal models also implicated that rhGH might improve NAFLD [11–13], and a clinical trial suggested that rhGH treatment might have benefits to reduce liver fat content in young adults with obesity and NAFLD [14]. However, to best of our knowledge, very few of studies used children as their experimental subjects, and there is no report focusing on the application of rhGH treatment to NAFLD with normal pituitary function in childhood obesity.

We have reported significant reductions in GH and IGF-1 in obese children with NAFLD [15, 16]. Furthermore, a recent study based on small sample clinical trials have confirmed that rhGH treatment can improve body mass index (BMI) and lipid metabolism without affecting glucose metabolism in obese children [17, 18]. However, whether rhGH treatment can improve NAFLD in obese children has never been investigated. In the present study, a randomized, open-label design was used to evaluate the effects of 6 months of rhGH administration in boys with obesity and NAFLD.

## Materials and methods

### Study design and participants

We conducted a randomized, open-label, 6-month clinical trial at the Department of Pediatrics, the Second Hospital of Shandong University between August 2018 and August 2020. The study was approved by the Ethics Committee of the Second Hospital of Shandong University and the study was registered with Clinical Trials.gov (ChiCTRIPR-17011267). Written informed consents have been signed by all participants and their parents. All methods were carried out in accordance with relevant guidelines and regulations.

Subjects were recruited from the Department of Pediatrics of the Second Hospital of Shandong University. A total of 172 boys with obesity and NAFLD were screened as participants. Inclusion criteria: boys with obesity and NAFLD aged 8–16 years. Exclusion criteria included: 1) secondary obesity, such as hypothyroidism, Cushing’s disease or hypothalamic obesity; 2) positive hepatitis B surface antigen, positive hepatitis C antibodies or other liver disease such as Wilson’s disease or autoimmune hepatitis etc.; 3) diabetes, hypothalamus-pituitary diseases or other disease condition known to affect the GH axis; 4) children who ever have had alcohol intake, smoked or used drugs which may influence liver function, glucose, lipid metabolism or weight within the 3 months prior to study; 5) serious infection, systemic disease, and other chronic wasting illnesses; 6) short stature or growth velocity is less than 5 cm/year. Finally, 100 boys with obesity and NAFLD were assessed according to the criteria of this study, and 72 obese adolescents met inclusion criteria and were selected as our final subjects.

Obesity was defined by body mass index (BMI) above the 95-percentile based on the Chinese national reference [19]. The diagnosis of NAFLD is estimated by ultrasound scan, and the calculation was made according to the criteria of The Chinese National Workshop on Fatty Liver and Alcoholic Liver Disease for the Chinese Liver Disease Association [20]. NAFLD was diagnosed upon detection of the presence of at least two of following three characteristic echo patterns [20]: 1) the presence of increased liver echogenicity; 2) diffuse hyperechogenicity of the liver relative to the kidneys; 3) poor visualization of the diaphragm or intrahepatic vessels.

### Protocol

**Fig. 1:**
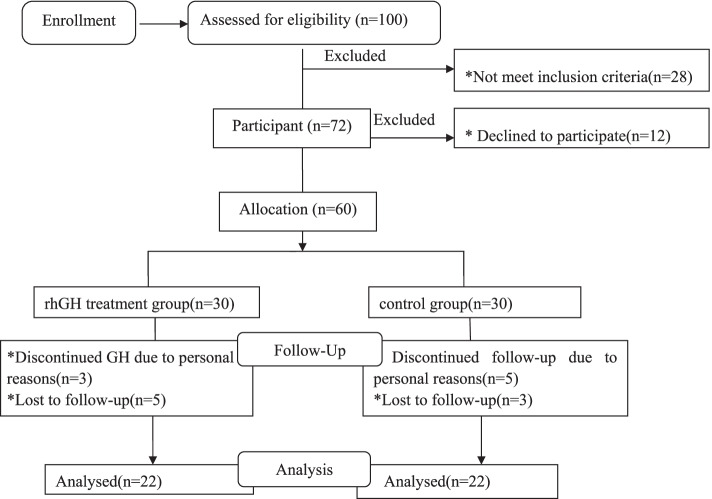
Flow chart of the study

Participant flow is shown in Fig. 1. Seventy two boys met the criteria and were selected as participators in the study, but 12 children declined to participate. Finally, sixty children and their parents agreed to participate in this clinical trial, then participants were randomized in a 1:1 ratio to recombinant human growth hormone (rhGH) treatment group or no treatment group. The study was 6 months in duration, with visits at baseline, 3 months and 6 months for all subjects. At each visit, the same endocrinologist will be given professional diet and exercise guidance. The guidance of dietary and exercise was according to the guideline presented by Styne et al. [21].

Anthropometric measurements, liver function, endocrine hormone levels, inflammatory factors and glucose and lipid metabolism indicators, abdominal ultrasound as well as imaging parameters were performed in all subjects in the baseline. After baseline evaluation, rhGH treatment group subjects received rhGH therapy (Changchun JinLei SaiZeng Co.) for 6 months. Daily dose of GH was 0.1 IU/kg (0.033 mg/kg). GH doses were adjusted based on weight levels and IGF-1 level at these visits. Anthropometric measurements, laboratory examinations (thyroid function, IGF-1, C reactive protein (CRP), liver function, lipid profile, glucose metabolism indicators, hemogram, and urinalysis) were performed for all visits. Abdominal ultrasound and oral glucose tolerance tests were repeated at the interval of 6 months.

During the follow-ups, eight subjects withdrew for personal reasons (3 in the rhGH group and 5 in the no treatment group) and eight subjects were lost in follow-ups (5 in the rhGH group and 3 in the non-treated group). Finally, 44 subjects completed the 6 months’ visit (22 in the rhGH group and 22 in the no treatment group).

In this study, anthropometric measurements included weight, height and pubertal stages. Weight was measured in the morning, with all subjects wearing light clothes and no shoes. It had nearest 0.1 kg errors using an electronic scale. Standing height was measured using a wall-mounted stadiometer in the morning to the nearest 0.1 cm. BMI (body mass index) was calculated from the ratio between weight and the quadratic term of height (kg/m^2^). BMI was expressed as BMI standard deviation scores (BMI SDS) based on normative values for Chinese children [19].The stage of puberty was assessed by physical examinations according to Tanner [22].

Fasting blood samples were obtained from all subjects after an overnight fast to measure the following parameters in baseline: CRP, alanine aminotransferase (ALT), aspartate aminotransferase (AST), gamma glutamyl transferase (GGT), total cholesterol (TC), high density lipoprotein cholesterol (HDL-C), low density lipoprotein cholesterol (LDL-C), triglycerides (TG), uric acid (UA), fasting blood glucose (FBG) and fasting insulin. All boys with obesity have perfectly performed the oral glucose tolerance test (1.75 g/kg, with the maximum dose of less than 75 g) in baseline and 6 months. We used homeostasis model assessment of insulin resistance (HOMA-IR) to represent insulin resistance, which was calculated as fasting insulin × fasting glucose/22.5 [23]. We also calculated whole body insulin sensitivity index (WBISI) for a more comprehensive assessment of insulin sensitivity in baseline and 6 months. WBISI = 10,000/square root of [fasting glucose × fasting insulin{μIU/mL}] × [mean glucose{mg/dL} × mean insulin during oral glucose tolerance test {μIU/mL}] [24].

Endocrine factors included IGF-1, free triiodothyronine (FT3), free thyroxine (FT4), thyroid-stimulating hormone (TSH), adrenal corticotropic hormone (ACTH), cortisol (COR), luteinizing hormone (LH) and follicle-stimulating hormone (FSH), which were also taken in the morning after a 12 h overnight fast.

The liver ultrasonographic was performed for all subjects in the morning after an overnight fast by a single trained and experienced operator using a 3.5–5 MHz transducer (Voluson E8, GE Healthcare, Austria) in baseline and after 6 months follow-up. Hypothalamic pituitary magnetic resonance imaging scans were also performed in all boys with obesity in baseline period.

### Statistical analysis

All variables were assessed for normality based on the method of Shapiro–Wilk test. Variables were expressed as mean ± standard deviation (SD) or median and range for normally distributed data or skewed distributed variables. Independent t-test was carried out for normally distributed data and skew distributed variables which can be log transformed to normally distributed variables. Skewed distributed variables which could not be transformed to normally distributed variables, were analyzed with the Mann–Whitney U-test. Categorical variables were compared by the chi-square test. Pearson or Spearman correlations were used as appropriate to determine associations between variables that were or were not normally distributed. Statistical analyses were performed by Statistical Package for Social Sciences software for windows version 20.0 (SPSS Inc. Chicago, USA).

## Results

Forty-four boys with obesity and NAFLD (11.76 ± 1.67 year) have completed the clinical trials. Clinical characteristics, inflammatory factors, metabolic factors and endocrine factors were similar between rhGH group and control group in baseline (Tables 1 and 2).

**Table 1:** Clinical characteristics of all obese with NAFLD children

Variable	rhGH group (*n* = 22)	control group (*n* = 22)	*p* value
Age (yr)	12.04 ± 1.56	11.48 ± 1.78	0.272
Prepuberty/Puberty, (n)	16/6	15/7	0.741▲
BMI SDS	2.56 ± 0.65	2.75 ± 0.61	0.321
CRP(mg/L)	1.75 ± 1.06	2.06 ± 1.22	0.378
ALT(U/L)	24.50(14.75**,** 46.00)	26.53(17.00**,** 60.25)	0.452▲
AST(U/L)	25.50(22.00**,** 29.75)	24.50(21.50**,** 34.25)	0.953▲
GGT(U/L)	21.50(17.00**,** 30.00)	28.00(21.00**,** 31.75)	0.09▲
TC(mmol/L)	4.57 ± 0.60	4.46 ± 0.97	0.654
HDL-C (mmol/L)	1.24(1.07**,** 1.40)	1.20(1.07**,** 1.37)	0.717#
LDL-C (mmol/L)	2.71 ± 0.45	2.68 ± 0.79	0.870
TG(mmol/L)	1.32(0.84**,** 1.87)	1.21(0.92**,** 1.58)	0.988#
UA(μmol/L)	381.05 ± 112.52	370.90 ± 68.55	0.720
Insulin(uIU/mL)	24.13 ± 11.41	25.91 ± 13.01	0.631
FBG(mmol/L)	5.17 ± 0.39	5.18 ± 0.38	0.972
HOMA-IR	5.58 ± 2.79	5.99 ± 3.13	0.648
WBISI	3.22 ± 1.16	2.84 ± 1.52	0.349
HbA1c (%)	5.68 ± 0.24	5.66 ± 0.34	0.839

^#^ *p* value reported for log transformed values, but values in the table represent as the median (interquartile range) ▲Mann–Whitney U test or chi square test

^*^ *p* < 0.05

*Abbreviations*: *BMI SDS* body mass index standard deviation scores, *CRP* C reactive protein, *ALT* Alanine aminotransferase, *AST* Aspartate aminotransferase, *GGT* Gamma-glutamyl transferase, *TC* Total cholesterol, *HDL-C* High density lipoprotein cholesterol, *LDL-C* Low density lipoprotein cholesterol, *TG* Triglycerides, *UA* Uric acid, *FBG* Fasting blood glucose, *HOMA-IR* Homeostasis model assessment of IR, *WBISI* Whole body insulin sensitivity index, *HbA1c* Glycosylated haemoglobin

**Table 2:** Endocrine factors of all obese with NAFLD children

Variable	rhGH group (*n* = 22)	control group (*n* = 22)	*p* value
IGF-1(ng/ml)	223.59 ± 94.82	279.23 ± 128.19	0.109
FT3 (pmmol/L)	6.20 ± 0.63	6.17 ± 0.43	0.816
FT4 (pmmol/L)	16.38 ± 2.31	15.65 ± 2.04	0.273
TSH (μU/mL)	2.77 ± 1.34	2.96 ± 1.05	0.611
ACTH (mmol/L)	24.31 ± 15.33	30.44 ± 13.27	0.163
COR(pg/mL)	355.54 ± 167.14	333.86 ± 136.94	0.640
LH(mIU/ml)	1.84(0.62**,** 3.34)	0.84(0.62**,** 2.65)	0.519#
FSH(mIU/ml)	4.29 ± 2.36	3.20 ± 2.04	0.110
Estradiol (pg/ml)	12.98(6.08**,** 30.85)	13.55(5.79**,** 24.72)	0.519#
Testosterone (ng/ml)	0.29(0.10**,** 0.75)	0.30(0.13**,** 0.95)	0.870#

^#^ *p* value reported for log transformed values, but values in the table represent as the median (interquartile range)

^*^ *p* < 0.05

*Abbreviations*: *IGF-1* Insulin-like growth factor 1, *FT3* Free Friiodothyronine, *FT4* Free thyroxine, *TSH* Thyroid-stimulating hormone, *ACTH* Adrenal corticotropic hormone, *COR* Cortisol, *LH* Luteinizing hormone, *FSH* Follicle-stimulating hormone

### Changes in IGF-1 levels after 3 months and 6 months of rhGH treatment

**Fig. 2:**
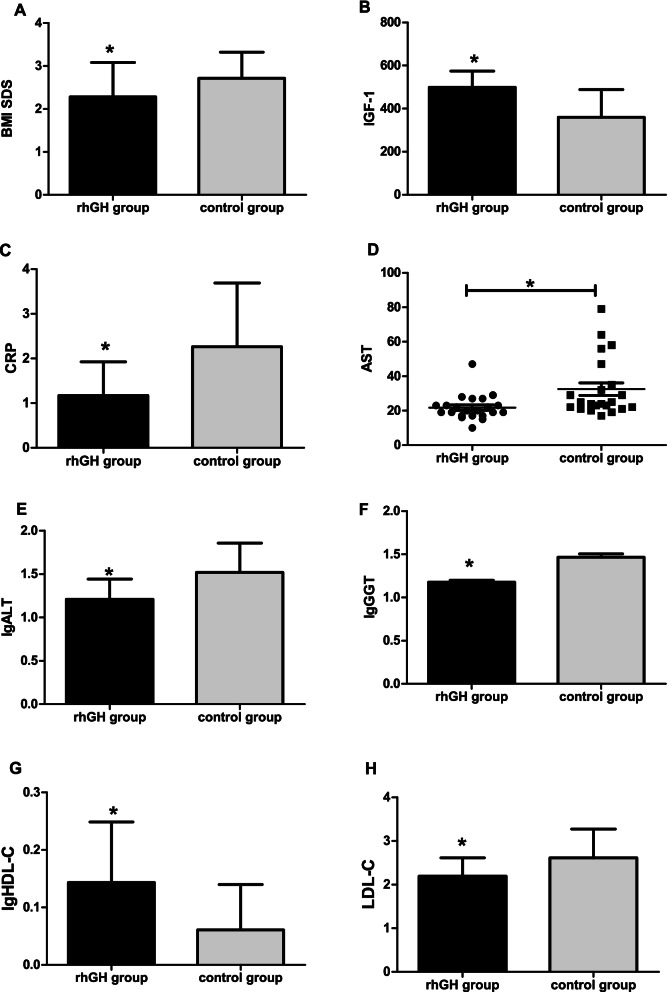
After 6 months, change in BMI SDS, IGF-1, CRP, lgALT, AST, lgGGT, lgHDL-C and LDL-C in rhGH treatment group compared with untreated control group. * *p* < 0.05 vs control group

IGF-1 in the rhGH treatment group and control group at baseline were 223.59 ± 94.82 and 279.23 ± 128.19 ng/ml (*p* = 0.109), respectively (see Table 2). After 3 months’ visit, IGF-1 in the rhGH group was significantly higher than it in the control group (473.36 ± 159.12 vs. 355.86 ± 126.98 ng/ml, *p* = 0.01) (see Table 3). At the 6th months, IGF-1 increased significantly during rhGH treatment, in comparison with the control group (582.45 ± 133.00 vs. 359.64 ± 129.00 ng/ml, *p* < 0.001) (see Table 4, Fig. 2B).

**Table 3:** Change in variables in obese with NAFLD children treated with GH vs control group for 3 months

Variable	rhGH group (*n* = 22)	control group (*n* = 22)	*p* value
BMI SDS	2.37 ± 0.69	2.66 ± 0.54	0.138
IGF-1(ng/ml)	473.36 ± 159.12	355.86 ± 126.98	0.01*
CRP(mg/L)	1.24 ± 0.80	2.07 ± 1.14	0.008*
ALT(U/L)	18.25(13.50**,**27.75)	21.00(16.75**,** 42.25)	0.113▲
AST(U/L)	21.50(19.88**,**28.75)	23.00(20.00**,** 30.50)	0.430▲
GGT(U/L)	14.00(12.75**,**17.75)	24.00(20.00**,** 33.25)	< 0.001*▲
TC(mmol/L)	3.99 ± 0.59	4.15 ± 0.76	0.432
HDL-C (mmol/L)	1.41 ± 0.30	1.12 ± 0.20	0.001*
LDL-C (mmol/L)	2.17 ± 0.42	2.49 ± 0.53	0.033*
TG(mmol/L)	0.90(0.71**,** 1.52)	1.05(0.82**,** 1.38)	0.578#
UA(μmol/L)	423.96 ± 97.51	376.64 ± 78.65	0.084
Insulin(uIU/mL)	31.16(21.28**,**36.75)	17.04(14.43**,** 28.46)	0.086#
FBG(mmol/L)	5.34 ± 0.31	5.25 ± 0.40	0.393
HOMA-IR	7.34(5.16**,** 8.76)	3.99(3.02**,** 6.94)	0.078#
HbA1c (%)	5.62 ± 0.35	5.62 ± 0.30	1#

^#^ *p* value reported for log transformed values, but values in the table represent as the median (interquartile range) ▲Mann–Whitney U test

^*^ *p* < 0.05

*Abbreviations*: *BMI SDS* Body mass index standard deviation scores, *IGF-1* Insulin-like growth factor 1, *CRP* C reactive protein, *ALT* Alanine aminotransferase, *AST* Aspartate aminotransferase, *GGT* Gamma-glutamyl transferase, *GH* Growth hormone, *TC* Total cholesterol, *HDL-C* High density lipoprotein cholesterol, LDL-C Low density lipoprotein cholesterol, *TG* Triglycerides, *UA* Uric acid, *FBG* Fasting blood glucose, *HOMA-IR* Homeostasis model assessment of IR, *HbA1c* Glycosylated haemoglobin

**Table 4:** Change in variables in obese with NAFLD children treated with GH vs control group for 6 months

Variable	rhGH group (*n* = 22)	control group (*n* = 22)	*p* value
BMI SDS	2.28 ± 0.80	2.71 ± 0.61	0.049*
IGF-1(ng/ml)	582.45 ± 133.00	359.64 ± 129.00	< 0.001*
CRP(mg/L)	1.17 ± 0.76	2.26 ± 1.43	0.003*
ALT(U/L)	15.00(10.90**,**22.50)	28.00(21.00**,** 54.25)	0.001*#
AST(U/L)	20.00(17.00**,**24.00)	24.50(21.75**,** 38.00)	0.004*▲
GGT(U/L)	14.50(13.75**,**18.25)	28.50(21.00**,** 36.00)	< 0.001*#
TC(mmol/L)	4.15 ± 0.64	4.30 ± 0.94	0.538
HDL-C (mmol/L)	1.30(1.23**,** 1.70)	1.15(1.01**,** 1.23)	0.005*#
LDL-C (mmol/L)	2.19 ± 0.42	2.61 ± 0.66	0.016*
TG(mmol/L)	1.06(0.72**,** 1.36)	1.05(0.73**,** 1.55)	0.589#
UA(μmol/L)	420.45 ± 85.38	397.82 ± 84.25	0.381
Insulin(uIU/mL)	25.19(17.33**,**41.33)	20.74(15.95**,** 28.63)	0.151#
FBG(mmol/L)	5.50 ± 0.44	5.36 ± 0.52	0.328
HOMA-IR	6.27(4.15**,** 9.14)	5.28(3.55**,** 6.63)	0.115#
WBISI	2.82 ± 1.21	3.01 ± 1.25	0.613
HbA1c (%)	5.66 ± 0.20	5.64 ± 0.32	0.866

^#^ *p* value reported for log transformed values, but values in the table represent a back transformation to the original▲Mann–Whitney U test

^*^ *p* < 0.05

*Abbreviations*: *BMI SDS* body mass index standard deviation scores, *IGF-1* Insulin-like growth factor 1, *CRP* C reactive protein, *ALT* alanine aminotransferase, *AST* aspartate aminotransferase, *GGT* Gamma-glutamyl transferase, *GH* growth hormone, *TC* total cholesterol, *HDL-C* High density lipoprotein cholesterol, *LDL-C* Low density lipoprotein cholesterol, *TG* triglycerides, *UA* Uric acid, *FBG* fasting blood glucose, *HOMA-IR* homeostasis model assessment of IR, *WBISI* Whole body insulin sensitivity index, *HbA1c* Glycosylated haemoglobin

### Changes in CRP levels after 3 months and 6 months of rhGH treatment

At baseline, the levels of CRP in the rhGH treatment group were similar to that of controls (1.75 ± 1.06 and 2.06 ± 1.22 mg/L respectively, *p* = 0.378) (see Table 1). At the 3rd month, participants in the rhGH group had a decrease in CRP compared to those receiving no treatment (1.24 ± 0.80 vs. 2.07 ± 1.14 ng/ml, *p* = 0.008) (see Table 3). At the 6th month, the rhGH group showed a significant decrease in CRP than in control group (1.17 ± 0.76 vs. 2.26 ± 1.43 ng/ml, *p* = 0.003) (see Table 4, Fig. 2C).

### Effects of GH administration on liver function and liver ultrasound

No differences were observed between rhGH group and the control group in AST, ALT and GGT at baseline (see Table 1). At the 3rd month, there were also no significant differences in AST and ALT between rhGH group and the control group, but GGT decreased in the GH group compared with the control group (*p* < 0.001) (see Table 3). At the 6th month, AST, ALT and GGT decreased significantly in the treatment group compared with in control group (*p* = 0.001, *p* = 0.004, *p* < 0.001) (see Table 4, Fig. 2D, E, F).

At the 6 months, liver ultrasonography has been done for all subjects to reassess the presence of NAFLD. In the rhGH treatment group, six subjects’ liver steatosis disappeared. In the control group, there were also 2 subjects without abnormal liver ultrasound. The differences between the two groups were not statistically significant (*p* = 0.24).

### Effects of GH administration on BMI SDS

BMI SDS of rhGH group subjects was similar to that of controls at baseline (2.56 ± 0.65 and 2.75 ± 0.61 respectively, *p* = 0.321) (see Table 1). At the 3th month, BMI SDS remained no significant difference between the two groups (2.37 ± 0.69 vs. 2.66 ± 0.54, *p* = 0.138) (see Table 3). At the 6th month, the treatment group had a greater decrease in BMI SDS in comparison with that of the control group (2.28 ± 0.80 vs. 2.71 ± 0.61, *p* = 0.049) (see Table 4, Fig. 2A).

### Effects of GH administration on cardiovascular metabolic parameters

At baseline, TC, HDL-C, LDL-C, TG and UA of the rhGH treatment group did not differ from the control group (Table 1). At the 3rd month, subjects in the rhGH group had significant increase in HDL-C compared with the control group (1.41 ± 0.30 vs. 1.12 ± 0.20 mmol/L, *p* = 0.001) (see Table 3), and the treatment group also have greater reductions in LDL-C comparing with controls (2.17 ± 0.42 vs. 2.49 ± 0.53 mmol/L, *p* = 0.033) (see Table 3). At the 6 month, HDL-C was also significantly higher than that of controls (*p* = 0.005) (see Table 4, Fig. 2G), and LDL-C of rhGH group subjects was significantly lower than the controls (2.19 ± 0.42 vs. 2.61 ± 0.66 mmol/L, *p* = 0.016) (see Table 4, Fig. 2H). TC, TG and UA had no significant differences between the rhGH treatment and the control group at the 3rd month and 6th month (see Tables 3 and 4).

### Effects of GH administration on glucose metabolism

**Fig. 3:**
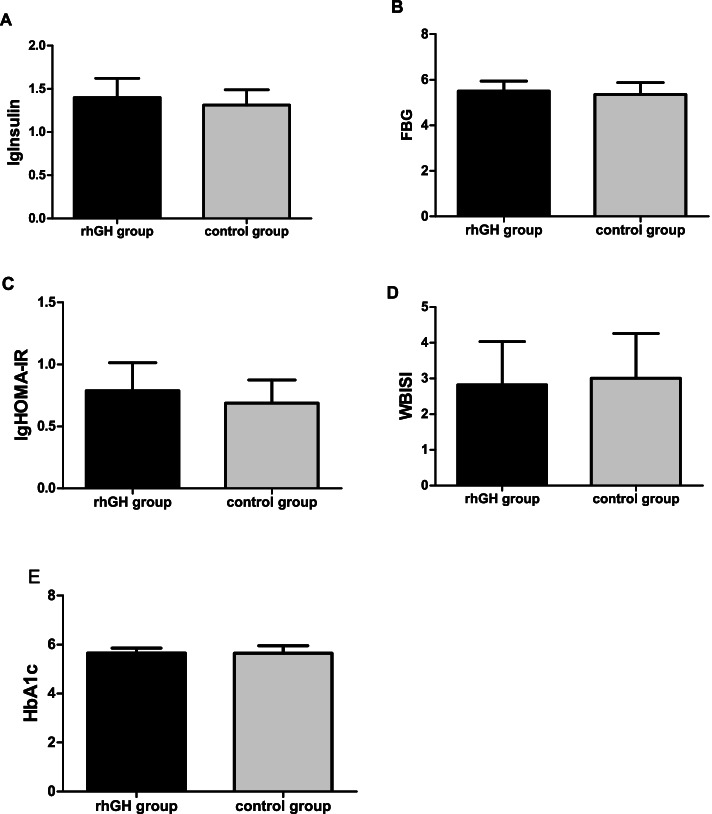
After 6 months, change in glucose metabolism in rhGH treatment group compared with untreated control group. * *p* < 0.05 vs control group

At baseline, there were no significant differences in insulin, FBG, HOMA-IR, WBISI, Glycosylated haemoglobin (HbA1c) between rhGH treatment group and control group (see Table 1). After 6 months of treatment and follow-ups, no significant effects were elicited by rhGH treatment on insulin, FBG, HOMA-IR, WBISI, HbA1c (see Tables 3, 4 and Fig. 3).

### Relationships between change in IGF-1 and other indexes for all subjects at 6 months

After the 6 months’ visit, we performed the correlation analysis between IGF-1 and other indexes for all subjects. IGF-1 was negatively associated with AST (*r* = –0.351, *p* = 0.019), ALT (*r* = –0.405, *p* = 0.006), GGT (*r* = –0.424, *p* = 0.004), CRP (*r* = –0.484, *p* = 0.001), BMI SDS (*r* = –0.372, *p* = 0.001), and positively associated with HDL-C (*r* = 0.366, *p* = 0.015), FBG (*r* = 0.368, *p* = 0.014), HOMA-IR (*r* = 0.307, *p* = 0.043). In addition, IGF-1 levels were not significantly associated with TC, TG, LDL-C, UA, insulin and HbA1c.

### Side effects

There were no serious adverse events related to study participation. At the end of the follow-ups, there were 6 participants in the treatment group and the control group had FBG above 6 mmol/L (3 in the rhGH group and 3 in the control group). But none of them exceeded 7 mmol/L, and no subject had a glucose level > 11.1 mmol/L at the 6 month’s oral glucose tolerance test. The HbA1c was below 6.5% of all participants in the treatment group and the control group. Two subjects in the rhGH group experienced the temporary hypothyroidism, which was found in the follow-up of 3 months of treatment or 6 months of rhGH treatment respectively. The thyroid function returned to normal after levothyroxine treatment and was under continuous follow-up. No subjects complained of headaches or oedema.

## Discussion

This study serves as an early attempt to demonstrate that rhGH treatment exerts beneficial effects on hepatic aminotransferase and markers of cardiovascular risk in boys with obesity and NAFLD without organic hypothalamic/pituitary disease. In the present study, we show that GH treatment for 6 months significantly increased IGF-1 levels, reduced serum liver enzyme, accompanied by the reductions of CRP, BMISDS and the improvements of the lipid profile, whereas no effect was observed on glucose metabolism. However, after 6 months’ rhGH treatment, there was no statistically significance in the outcome of NAFLD in these two groups.

In this study, a significant reduction in serum GGT was observed in the GH-treated boys after 3 months. Furthermore, AST, ALT and GGT were all marked after 6 months’ treatment in rhGH group comparing with those in the control group. Although no statistically significant change or recovery of NAFLD which was measured by liver ultrasound has been observed, rhGH treatment was statistically efficacious for improving serum liver enzyme levels after 6 months. Collectively, we suggest a possible positive effect of rhGH on liver enzyme in boys with obesity. However, we were also concerned that although rhGH therapy of liver enzymes was statistically significant, the median ALT level moved from the normal range to the normal range. Given the high cost and possible side effects of rhGH treatment, there is still a long way to go before it can be used in the treatment of NAFLD and liver enzymes.

The potential pathophysiology mechanism of why short-term rhGH treatments would be effective for children with NAFLD is not yet completely can be explained as follows. First, IGF-1 has been shown to be a protective factor for resisting NAFLD and plays an essential role in the prevention of NAFLD [25], rhGH may improve NAFLD by increasing IGF-1. Second, previous studies have shown that rhGH treatment can improve BMI and lipid metabolism [17, 18]. GH may further improve hepatic steatosis by improving lipid metabolism [13]. Third, GH may ameliorat hepatocyte steatosis by suppressing denovo lipogenesis via carbohydrate responsive element-binding protein and fatty acid synthase down-regulation [26].

Serum CRP is the most validated inflammatory marker and has been shown to be a sensitive independent marker for the occurrence and development of NAFLD [27–29]. We observed a significant decrease of CRP level in the GH group comparing with controls in our study of boys with obesity and NAFLD. The results indicated that GH replacement therapy could improve chronic inflammatory state. In contrast to the results by prior study, Pan et al. [14] reported no significant changes in CRP after 6 months of GH administration in young adults with obesity and NAFLD. One possible reason is that these studies reflect differences in study population and the dose of GH.

As an anabolic hormone, GH has a clear lipolysis effect, especially for visceral fat [30]. Previous studies have shown that GH replacement in patients with obesity reduced BMI and improved the lipid profile and other cardiovascular risk factors [17, 18, 31]. Our study found the similar beneficial effect of GH in boys with obesity and NAFLD. Specifically, our findings demonstrated that GH could reduce BMI SDS and improve lipid profiles by decreasing LDL-C, and increasing HDL-C in boys with NAFLD and obesity. In this study, we found that there were no significant changes in TG during GH therapy, which is in accordance with previous findings based on the subjects of obese children [17] and adults [32]. In addition, we did not observe evidences of GH treatment in UA in boys with NAFLD and obesity which had never been tested in prior research related to GH treatment.

Alterations in the thyroid function have been reported following rhGH treatment in children with [33, 34] and without GH deficiency [17, 18, 34]. The possible mechanism of hypothyroidism is rapid growth during rhGH treatment, compensatory deficiency in thyroid function occurred [34]. In our study, two subjects experienced hypothyroidism in the rhGH group. This result is also consistent with previous studies on the application of rhGH to obese children [17, 18].

There are several limitations in this study. First, we did not perform liver biopsies as an evaluation criterion for NAFLD in our subjects. Liver biopsy was regarded as one of the gold standards for NAFLD diagnosis, but it is not recommended being performed in children because it is an invasive method [35]. The second limitation is the relatively small sample size used in this study and the participants were all boys. Future studies can consider to increase the number of participants and conduct prospective studies to further validate our results. Thirdly, two-way ANOVA is a more appropriate statistical method for our research design, but our data did not meet the requirements of applying two-way ANOVA analysis. Future studies can apply a two-way ANOVA analysis if the data all normally distributed variables to provide more comprehensive and convincing evidences relating to our research questions. Another limitation was the relatively high drop-out rate of the study. The epidemic of COVID-19 is an important reason for the increase in the dropout rate. Another reason of high dropout rate is that trials of obesity children therapies face the serious problem that children always cannot adhere to long-term treatment and also the problem of the lack of parents’ attentions to childrens’ obesity. However, the dropout rate was lower than or comparable to other obesity studies and similar [36, 37] in the GH and control groups. Finally, this study is a short-term clinical trial thus the sample size is relatively small compared with prior studies based on long-term clinical trials. It remains to be determined whether GH can be used in the treatment of NAFLD. Further studies are necessary to further explain the underlying mechanisms and develop potential therapeutic effect of rhGH for NAFLD.

## Conclusion

In conclusion, the results indicated that the short-term rhGH treatment might be beneficial to liver enzyme and it could improve obesity-related other cardiovascular and metabolic complications in boys with obesity and NAFLD. No serious adverse reactions were found during the treatment. Further investigation is necessary to clarify the precise mechanisms among GH, IGF-1and NAFLD, and whether or not long-term GH treatment can improve the NAFLD and the specific dose of GH also needs to be determined by further studies with larger samples.

## Data Availability

The datasets used and/or analysed during the current study are available from the corresponding author on reasonable request.

## Abbreviations

NAFLD: Non-alcoholic fatty liver disease
GH: Growth hormone
IGF-1: Insulin-like growth factor 1
BMI: Body mass index standard
CRP: C reactive protein
BMI SDS: Body mass index standard deviation scores
ALT: Alanine aminotransferase
AST: Aspartate aminotransferase
GGT: Gamma-glutamyl transferase
TC: Total cholesterol
HDL-C: High density lipoprotein cholesterol
LDL-C: Low density lipoprotein cholesterol
TG: Triglycerides
UA: Uric acid
FBG: Fasting blood glucose
HOMA-IR: Homeostasis model assessment of insulin resistance
WBISI: Whole body insulin sensitivity index
FT3: Free Friiodothyronine
FT4: Free thyroxine
TSH: Thyroid-stimulating hormone
ACTH: Adrenal corticotropic hormone
COR: Cortisol
LH: Luteinizing hormone
FSH: Follicle-stimulating hormone
HbA1c: Glycosylated haemoglobin
